# Magnetization States and Coupled Spin-Wave Modes in Concentric Double Nanorings

**DOI:** 10.3390/nano14191594

**Published:** 2024-10-02

**Authors:** Bushra Hussain, Michael G. Cottam

**Affiliations:** 1 Department of Natural Sciences, University of Michigan, Dearborn, MI 48197, USA; bhussai@umich.edu; 2 Department of Physics and Astronomy, University of Western Ontario, London, ON N6A 3K7, Canada

**Keywords:** spin waves, ferromagnetic nanorings, concentric double nanorings, dipole–dipole interactions, dipole-exchange modes

## Abstract

Concentric multiple nanorings have previously been fabricated and investigated mainly for their different static magnetization states. Here, we present a theoretical analysis for the magnetization dynamics in double nanorings arranged concentrically, where there is coupling across a nonmagnetic spacer due to the long-range dipole–dipole interactions. We employ a microscopic, or Hamiltonian-based, formalism to study the discrete spin waves that exist in the magnetic states where the individual rings may be in either a vortex or an onion state. Numerical results are shown for the frequencies and the spatial amplitudes (with relative phase included) of the spin-wave modes. Cases are considered in which the magnetic materials of the rings are the same (taken to be permalloy) or two different materials such as permalloy and cobalt. The dependence of these properties on the mean radial position of the spacer were studied, showing, in most cases, the existence of two distinct transition fields. The special cases, where the radial spacer width becomes very small (less than 1 nm) were analyzed to study direct interfaces between dissimilar materials and/or effects of interfacial exchange interactions such as Ruderman–Kittel–Kasuya–Yoshida coupling. These spin-wave properties may be of importance for magnetic switching devices and sensors.

## 1. Introduction

There has been much interest in studying finite-sized ferromagnetic structures with μm and nm dimensions for their intrinsic scientific properties and their broad range of applications, which include high-frequency data storage systems, logic systems, bio(sensors), microwave systems, as well as in various medical applications [1,2,3,4,5,6,7,8,9,10,11,12]. An increasing emphasis has been given to investigating the static and dynamic properties of ferromagnetic nanorings in particular, because they can exhibit various magnetic states, switching behavior, and unique spin dynamics [13,14,15,16]. These characteristics can be controlled by changing the geometry, composition and/or by application of an external static magnetic field. When the thickness of the rings is small compared to their inner and outer radii, they generally have two magnetic states, the vortex (or flux-closure) state and the onion (or bi-domain) state; these arise mainly due to competing effects between the dipole–dipole and exchange interactions in an individual ring, as well as the Zeeman energy of an applied field. A vortex state (VS) is favored at low applied magnetic field values with an abrupt transitioning to an onion state (OS) which occurs at a higher fields [14,15,17,18,19,20,21,22,23,24]. Various basic shapes of the individual rings (for example, disks and/or rings with either square, rectangular, circular, or elliptical cross sections) have been considered in these studies [18,25,26,27,28,29,30,31,32,33,34].

Compound ring structures of various types have also received attention. For example, magnonic crystal arrays of nanorings can be formed, either from a lateral periodic arrangement [35] or a vertical periodic stacking to produce a nanotube [36]. These are of significance due to their enhanced flexibility in controlling the spin dynamics as well as the creation of a greater range of metastable states of magnetic ordering [36,37,38,39]; this could make them suitable for, e.g., multi-state switches and high-density storage devices [4,7].

Here, we consider a different type of compound-ring geometry, consisting of multiple concentric ferromagnetic nanorings with intervening nonmagnetic spacers. Concentric ferromagnetic nanorings have been fabricated and studied experimentally to demonstrate their use as multi-bit storage devices for MRAM technology [40,41]. In other studies, micromagnetic simulations were performed to investigate the dynamic behavior of concentric rings as a function of their geometric parameters [42,43]. Furthermore, Sahu et al. [44] recently used micromagnetic simulations to explore the static and dynamic behavior of concentric multiple rings; specifically, the resonances found for a dynamic susceptibility were used to probe some of the excited spin-wave states. By contrast, in the work presented here, we explore the spin-wave (SW) properties of concentric double nanorings. We focus on a pair of concentric ferromagnetic nanorings, separated by a narrow nonmagnetic ring spacer in the same lateral plane. Compared with the single-nanoring case, we are introducing additional interfaces in the radial direction, which we show will give rise to novel field-dependent magnetic states. The long-range dipole–dipole interactions act between all spins within and between the nanorings (thus providing an inter-ring coupling), and they compete with the short-range exchange interactions, typically within each nanoring. By using a microscopic (or Hamiltonian-based) formalism, by analogy with previous work on other nanoring systems [45,46], we show that the different possible magnetic states, depending on the applied magnetic field, may involve either vortex states (with the same or opposite chirality) in both nanorings, a vortex state in one nanoring and an onion state in the other, or onion states in both nanorings. Hence, there are two separate magnetic transition fields involving these states, instead of just one transition field as for a single nanoring. Following a normal-mode calculation, the frequencies and relative amplitudes of all the discrete SW modes are calculated here in all three states of the concentric double nanoring system; these modes show distinctive changes in their behavior at the phase boundaries. By tailoring the composition and the wall thicknesses of the individual nanorings, as well as the intervening spacer thicknesses, the SW frequencies and their spatial amplitudes can be varied in a controlled fashion.

In general, the SW dynamics in single nanorings have been investigated experimentally using techniques such as Brillouin light scattering (BLS) and ferromagnetic resonance (FMR), for example [14,15,47]. On the theoretical side, both macroscopic (or continuum) and microscopic (or Hamiltonian-based) approaches have been successfully followed (see, e.g., [32,37,45,46,47,48,49,50]). Here, we conveniently employ the latter method.

The paper is organized as follows: Section 2 on materials and methods is where we describe the geometry, composition, and theory for the concentric double nanorings. The theory is given in two stages; the first part is applied to obtain the stable magnetization states in the two nanorings and, in the second part, we determine the SW frequencies and their spatial amplitudes. Section 3 for the results contains numerical examples obtained initially for single-component structures, where both nanorings are made of Py. Later, in the same section, we show numerical results for bicomponent concentric double nanorings, i.e., those where one ring is made of Py and the other one is Co, arranged in either order (Py-Co or Co-Py). Finally, in Section 4, we discuss and summarize the results, indicating some prospective extensions of this work.

## 2. Materials and Methods

**Figure 1:**
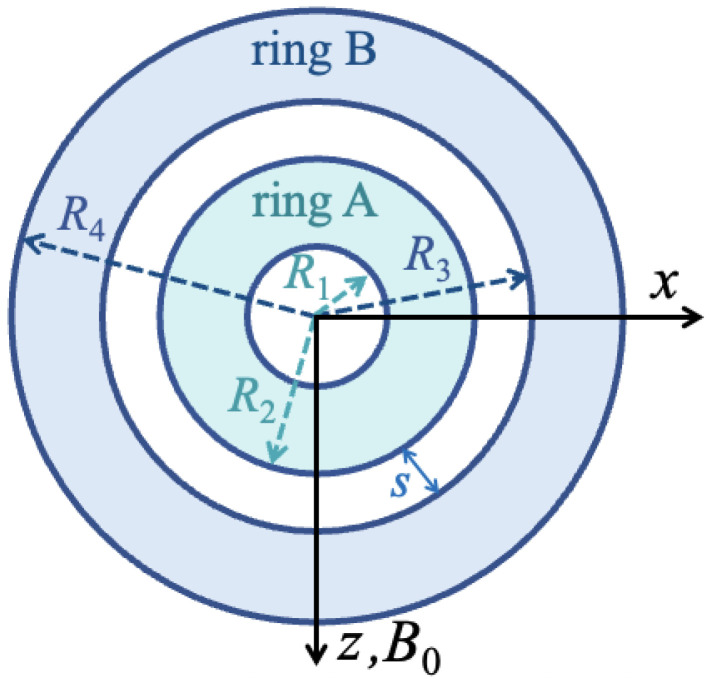
Schematic showing the geometry of thin concentric double nanorings in the xz plane, with the two magnetic rings labeled as A and B, which may be made of the same or different materials. The inner and outer radii of ring A are $R_{1}$ and $R_{2}$ and those of ring B are $R_{3}$ and $R_{4}$, respectively. In general, there is an intermediate nonmagnetic spacer *s* between A and B. The external magnetic field $B_{0}$ is applied along the *z* axis.

A double concentric nanoring was modeled with the geometry as shown in Figure 1. The inner and outer radii of the rings are labeled as $R_{1}$ and $R_{2}$ for ring A and $R_{3}$ and $R_{4}$ for ring B, respectively. Both nanorings are taken to have the same thickness *t* in the *y* direction, where $t\ll R_{l}$ with l=1,2,3,4. The radial spacer width *s* between the two rings (i.e., the difference between $R_{2}$ and $R_{3}$) can be varied to control the degree of magnetic coupling through the long-range dipolar interactions. In this work, we took the nanorings to be either composed of the same material, specifically both are Py, or different materials, chosen as Py and Co, to study their effects on the static and dynamic magnetizations.

These circular nanorings lie in the xz plane with an applied static magnetic field $B_{0}$ set along the *z* direction. In the calculations, we represented the magnetic material as a discrete cubic lattice with the length of sides *a*. The effective spins are taken to be at the center of each cell. From previous work, it is known that, to obtain a true description of the SW dynamics, it is reasonable to choose $a<a_{ex}$, where $a_{ex}$ is the exchange length of the material, which is of order 5–7 nm for both Py and Co. In our current calculations, we chose a= 3 nm, but we also checked the validity of our results at smaller values and found no significant difference at those lower values of *a*. Only the lattice sites lying within the nanorings (blue and cyan regions in Figure 1) were assigned a physical spin.

### 2.1. Theory for Equilibrium Configurations

The total microscopic spin Hamiltonian H for the system (see, e.g., [45]) given below includes contributions from the short-range Heisenberg-exchange interactions between nearest-neighbor sites, the long-range dipole–dipole interactions within and between the two nanorings, and the Zeeman-energy contribution from the external magnetic field $B_{0}$. Mathematically, we express

(1) $$H=-\frac{1}{2}\sum_{p,p^{\prime }}J_{p,p^{\prime }}S_{p}\cdot S_{p^{\prime }}+\frac{1}{2}{\left(g\mu_{B}\right)}^{2}\sum_{p,p^{\prime },\gamma ,\beta }D_{p,p^{\prime }}^{\gamma \beta }S_{p}^{\gamma }S_{p^{\prime }}^{\beta }-g\mu_{B}B_{0}\sum_{p}S_{p}^{z}.$$

Here, $S_{p}$ and $S_{p^{\prime }}$ are spin operators at spin sites *p* and $p^{\prime }$, respectively, *g* is the Landé factor, $\mu_{B}$ is the Bohr magneton, and γ and β are Cartesian components (*x*, *y*, or *z*). The Heisenberg-exchange term $J_{p,p^{\prime }}$ is nonzero only for nearest-neighbor spin sites, which typically will lie within the same nanoring when s>a for the spacer. The second term of Equation (1) is due to the magnetic dipole–dipole interactions where

(2) $$D_{p,p^{\prime }}^{\gamma \beta }=\frac{|r_{p,p^{\prime }}{|}^{2}\delta_{\gamma ,\beta }-3r_{p,p^{\prime }}^{\gamma }r_{p,p^{\prime }}^{\beta }}{|r_{p,p^{\prime }}{|}^{5}}$$

and $r_{p,p^{\prime }}=\left(x_{p}-x_{p^{\prime }},y_{p}-y_{p^{\prime }},z_{p}-z_{p^{\prime }}\right)$. The summations over *p* and $p^{\prime }$ in Equation (1) are over all distinct pair of sites (i.e., $p\neq p^{\prime }$) in the concentric double nanorings.

To study the SW dynamics in these thin nanorings, in which there can be multiple stable states, we first need to know the equilibrium (or static) spin orientations as a function of position in the system. These orientations can be determined in a mean-field approximation following [21] by using a total free-energy functional $\bar{E}$ at low temperatures. To obtain this quantity, we re-express the Hamiltonian by replacing each spin operator $S_{p}$ in Equation (1) with its classical vector $S(\sin\theta_{p}\cos\alpha_{p},\sin\alpha_{p}\sin\theta_{p},\cos\theta_{p})$, where $\theta_{p}$ represents the polar angle with respect to the *z* axis and $\alpha_{p}$ is the azimuthal angle with respect to the *x* axis. This yields

(3) $$\begin{array}{ccc} \bar{E} & = & -\frac{1}{2}S^{2}\sum_{p,p^{\prime }}J_{p,p^{\prime }}(\sin\theta_{p}\cos\alpha_{p}\sin\theta_{p^{\prime }}\cos\alpha_{p^{\prime }}+\sin\alpha_{p}\sin\theta_{p}\sin\alpha_{p^{\prime }}\sin\theta_{p^{\prime }}\\ & & +\cos\theta_{p}\cos\theta_{p^{\prime }})+\frac{1}{2}g^{2}\mu_{B}^{2}S^{2}\sum_{p,p^{\prime }}(D_{p,p^{\prime }}^{x,x}\sin\theta_{p}\cos\alpha_{p}\sin\theta_{p^{\prime }}\cos\alpha_{p^{\prime }}\\ & & +D_{p,p^{\prime }}^{x,y}\sin\theta_{p}\cos\alpha_{p}\sin\alpha_{p^{\prime }}\sin\theta_{p^{\prime }}+D_{p,p^{\prime }}^{x,z}\sin\theta_{p}\cos\alpha_{p}\cos\theta_{p^{\prime }}\\ & & +D_{p,p^{\prime }}^{y,x}\sin\alpha_{p}\sin\theta_{p}\sin\theta_{p^{\prime }}\cos\alpha_{p^{\prime }}+D_{p,p^{\prime }}^{y,y}\sin\alpha_{p}\sin\theta_{p}\sin\alpha_{p^{\prime }}\sin\theta_{p^{\prime }}\\ & & +D_{p,p^{\prime }}^{y,z}\sin\alpha_{p}\sin\theta_{p}\cos\theta_{p^{\prime }}+D_{p,p^{\prime }}^{z,x}\cos\theta_{p}\sin\theta_{p^{\prime }}\cos\alpha_{p^{\prime }}\\ & & +D_{p,p^{\prime }}^{z,y}\cos\theta_{p}\sin\alpha_{p^{\prime }}\sin\theta_{p^{\prime }}+D_{p,p^{\prime }}^{z,z}\cos\theta_{p}\cos\theta_{p})-g\mu_{B}B_{0}S\sum_{p}\cos\theta_{p}. \end{array}$$

Next, the components of the effective magnetic field acting on any spin site *p* in the nanoring system can be obtained using

(4) $$B_{\mathrm{eff}}^{\gamma }\left(p\right)=-\frac{1}{g\mu_{B}}\;\frac{\delta \bar{E}}{\delta S_{p}^{\gamma }}\;,$$

where γ=x,y, or *z*. We next find the equilibrium spin configurations by making an initial choice for the set of angles $\left\{\theta_{p},\alpha_{p}\right\}$ to approximately represent the ground state. In order to avoid the occurrence of unphysical local minima, we need to use several different starting configurations to obtain, eventually, the global ground state. If we had a single nanoring, for example, the starting configurations would be VS and OS (as in [21]). However, in the present case of a double nanoring other combinations of the simple VS and OS will become plausible possibilities. These may be VS in both nanorings (with the same or opposing chirality), OS in both, or VS in the inner ring and OS in the outer one or vice versa. For any of these starting configurations, the individual spin directions are determined with respect to the corresponding local effective field obtained using Equation (4), leading to a new set of angles. The process is repeated, typically for several thousands of these iterations, until convergence to an equilibrium stable state is achieved. Then, the *global* minimum is adopted for the equilibrium configuration corresponding to the value of the applied magnetic field. Finally, the process is carried out independently for all the other field values of interest.

### 2.2. Theory for Magnetization Dynamics

After determining the equilibrium spin configurations, we convert the initial global coordinates (x,y,z) to a new set of *local* coordinates (X,Y,Z), which are defined such that the new *Z* axis is aligned with the equilibrium direction at each spin site. Then, we find the dynamic SW properties by following the steps similar to those given in [51,52]. This involves applying a rotational transformation to convert the spin Hamiltonian (1) to the new set of axes. Next, it is convenient to transform to boson operators for a description of the SWs using the Holstein–Primakoff representation (see, e.g., [53,54,55]). The general form of the total boson Hamiltonian can be written as $H=H^{\left(0\right)}+H^{\left(1\right)}+H^{\left(2\right)}+H^{\left(3\right)}+...$, where $H^{\left(j\right)}$ represents a term with *j* boson operators. Here, $H^{\left(0\right)}$ is a constant and $H^{\left(1\right)}$ vanishes by symmetry. The terms with j>2 are of concern only when considering nonlinear effects and are ignored here. We are interested in the linearized (or non-interacting) SWs, and, so, use is only made of the bilinear term $H^{\left(2\right)}$ written as

(5) $$H^{\left(2\right)}=\sum_{p,p^{\prime }}\left\{C_{p,p^{\prime }}^{\left(2\right)}a_{p}^{†}a_{p^{\prime }}+C_{p,p^{\prime }}^{\prime \left(2\right)}a_{p}^{†}a_{p^{\prime }}^{†}+C_{p,p^{\prime }}^{\prime \left(2\right)*}a_{p}a_{p^{\prime }}\right\}.$$

Here, $a_{p}^{†}$ and $a_{p}$ are the boson creation and annihilation operators, respectively, associated with spin site *p*, and the coefficients $C_{p,p^{\prime }}^{\left(2\right)}$ and $C_{p,p^{\prime }}^{\prime \left(2\right)}$ are functions of the equilibrium angles and the parameters in the original Hamiltonian Equation (1) (see the appendix in [21]).

In a final step, we diagonalize the above Equation (5) using a generalized Bogoliubov transformation [51,52,56,57] to determine a set of SW modes. This step generates a dynamical block matrix defined by

(6) $$\left(\begin{array}{cc} C^{\left(2\right)} & 2C^{\prime (2)}\\ -2C^{\prime (2)*} & -{\tilde{C}}^{\left(2\right)} \end{array}\right),$$

where $C^{\left(2\right)}$ and $C^{\prime (2)}$ denote the N×N matrices provided by the above coefficients, the tilde denotes a transpose, and *N* is the number of spin sites in the double nanorings. There are *N* positive eigenvalues obtained from this matrix (6) that correspond to the physical SW frequencies. The remaining *N* negative eigenvalues are degenerate (in magnitude) and represent modes with the opposite sense of precession. The “diagonalized” form of the second-order Hamiltonian can formally be written as the sum over simple harmonic oscillators as

(7) $$H^{\left(2\right)}=\sum_{m=1}^{N}\omega_{m}\;b_{m}^{†}b_{m},$$

where $\omega_{m}$ represents the discrete SW frequencies with m=1,2,⋯,N being a mode or branch number. The quantities $b_{m}^{†}$ and $b_{m}$ are the diagonalized boson operators for the creation and annihilation of individual SW modes. From the eigenvectors of the matrix in Equation (6), we may obtain the spatially-dependent amplitudes, with phase information included.

## 3. Results

### 3.1. Single-Component Structures

Here, we took concentric double nanorings made of a single ferromagnetic material, choosing Py for both the inner and outer nanorings (labeled Py-Py), whereas, in the next section, we took two different materials (Co and Py) in our compound structure. We employed the values of magnetic parameters of Py, as used in a previous study (see, e.g., [58]) for other geometric Py samples: these are the exchange stiffness $D_{\mathrm{Py}}$ = 30.0 T nm^2^, the saturation magnetization $M_{s}$ is such that $4\pi \mu_{0}M_{s}$ = 0.115 T, and the gyromagnetic ratio $g\mu_{B}$ = 29.5 GHz T^−1^. The relation of these quantities to the parameters in the Hamiltonian (1) are given as $D=SJa^{2}/g\mu_{B}$, where *J* is the exchange interaction acting between the nearest neighbors only, and $M_{s}=g\mu_{B}S/a^{3}$. The effective lattice parameter *a* is selected to be less than the exchange correlation length $a_{ex}=\sqrt{D/4\pi M_{s}}$ (∼6 nm in Py), where D is related to the micromagnetic stiffness A by $D=2A/M_{s}$.

**Figure 2:**
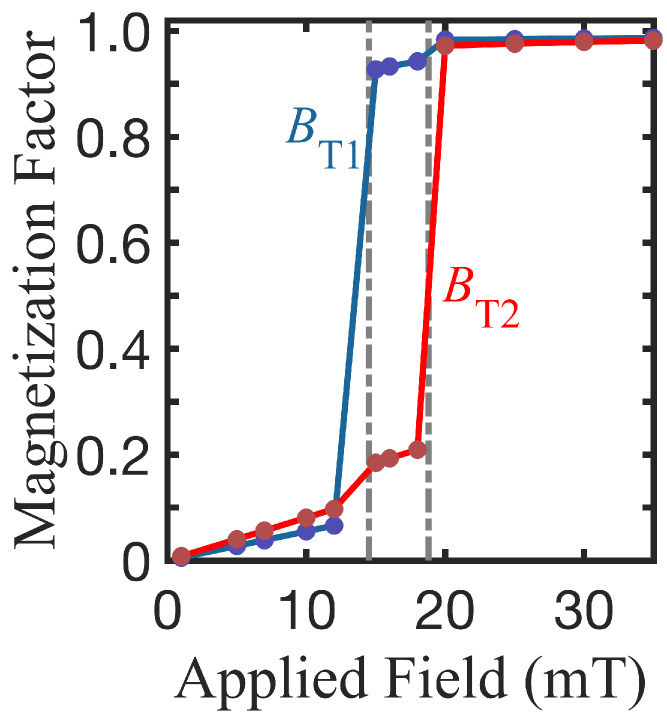
Magnetization factors versus applied magnetic field for a Py-Py nanoring with radii $R_{1}$ = 24 nm and $R_{2}$ = 45 nm for ring A and $R_{3}$ = 51 nm and $R_{4}$ = 102 nm for ring B. The blue and the red curves refer to the inner and outer nanorings, respectively. The two transition fields are shown as vertical dashed lines.

First, we present results for the plotted static and dynamic properties of concentric double Py-Py nanorings versus the mean radial spacer position $R_{m}=\left(R_{3}+R_{2}\right)/2$, ranging from 42 to 78 nm. Since there are many length variables in the nanoring system, we simplified the analysis by keeping as constants the overall dimensions of the nanoring structure by setting the inner radius $R_{1}$ and the outer radius $R_{4}$ to have the fixed values of 24 and 102 nm, respectively. Then, the spacer radial width $s=(R_{3}-R_{2})$ was kept constant while $R_{m}$ varied. For convenience we used the value of a= 3 nm in these calculations. In Figure 2, which displays the magnetization factors of rings A and B as a function of the applied magnetic field, we see that the switching between the VS and OS configurations occurs at different magnetic fields for the two nanorings. In this example (with $R_{m}=$ 48 nm), the first transition field labeled $B_{T1}$ for ring A is 15.1 mT and the second transition field labeled $B_{T2}$ for ring B is 21.0 mT.

**Figure 3:**
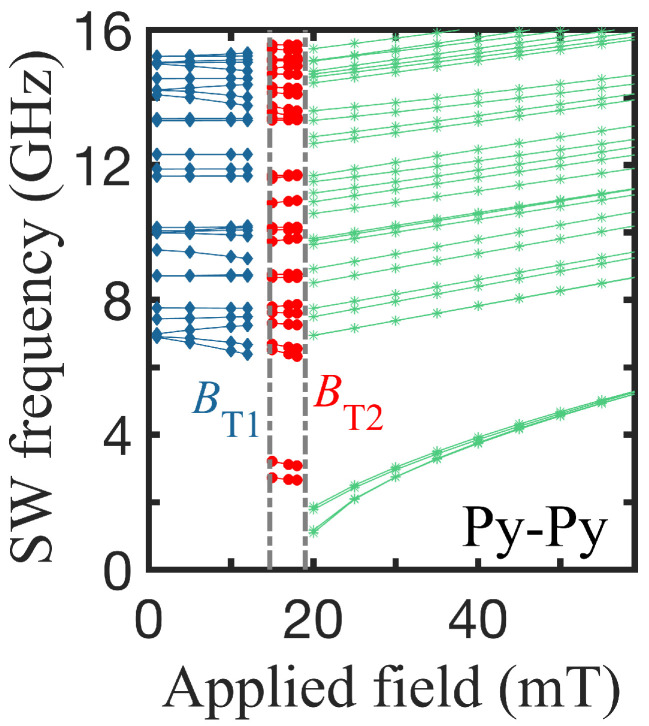
Several of the lowest SW frequencies versus the applied magnetic field showing the three stable regions for the same Py-Py nanorings, as used in Figure 2. There are two successive transition fields $B_{T1}$ and $B_{T2}$, giving the boundaries between the regions.

Since we were primarily interested in the dynamical properties of these concentric double nanorings, we show plots in Figure 3 of the SW frequencies as a function of the applied field $B_{0}$, using the same geometry as in Figure 2. As mentioned previously, there are three different regions of behavior for the discrete SW modes. In the first field region below $B_{T1}$, both rings are in VS (labeled VS-VS) with the same or opposite chirality, in the middle region (between the two transition fields), the ring A is in OS and ring B in VS (i.e., OS-VS), and, for fields above $B_{T2}$, both rings attain the OS as the stable state (labeled OS-OS). The three regions therefore show distinct dynamic features. For fields below $B_{T1}$ (in the VS-VS configuration), several of the SW modes vary in frequency as the field increases, whereas, above the first transition (for the OS-VS), the SW frequencies are lower for each mode compared to the VS-VS region. In the region above the second transition, the frequency values drop further but monotonically increase with the field, which is the effect of the switch to OS-OS as the Zeeman energy increases.There are abrupt changes in the SW frequencies at both phase boundaries linked to the switching of the magnetic ordering.

**Figure 4:**
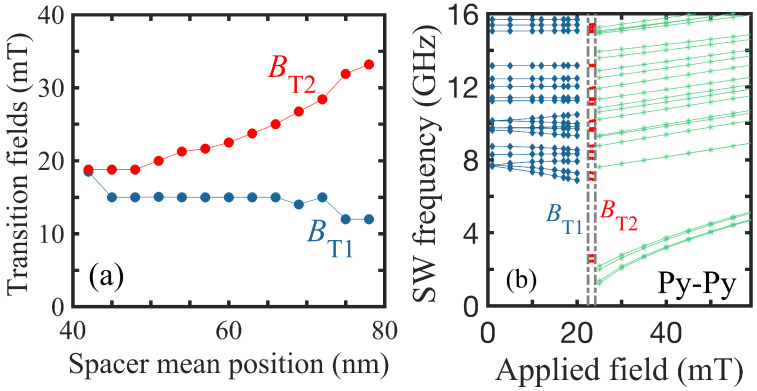
(**a**) Dependence of the transition fields $B_{T1}$ and $B_{T2}$ on mean radial position of the spacer for a Py-Py nanoring. The values of $R_{1}$ and $R_{4}$ are fixed at 24 nm and 102 nm, respectively. (**b**) Example showing the transition fields and lowest SW frequencies when the outer ring radius $R_{4}$ is changed to 87 nm. The other length parameters are as before in Figure 2.

Next, the variations found for the transition fields $B_{T1}$ and $B_{T2}$ when the mean spacer radius $R_{m}$ is changed in position are presented in Figure 4a. We see that, as $R_{m}$ is increased, the general trend is for $B_{T1}$ to decrease and for $B_{T2}$ to increase. Thus, the intermediate region where one ring is in VS and the other in OS becomes more favored energetically when the spacer moves outward. Interestingly, we find that the first transition field stays at a value almost independent of the spacer position, whereas the second transition field increases as the mean spacer position is increased (making the wall width of the inner ring increase while the wall width of the outer ring decreases). At small $R_{m}\approx 42$ nm in this case, our numerical results show that the vortex state in the inner part of the ring is sufficiently stable that a transition proceeds directly to an onion state (with $B_{T1}=B_{T2}$) when the applied field is increased.

To supplement the calculated data to include other choices for the radii of the rings, we show, in Figure 4b, an example where the outer radius $R_{4}$ is reduced to 87 nm, while the other radii are taken to have the same values as in Figure 2. This makes the radial widths of the two parts of the ring more comparable in value, and we see that the two transition fields have shifted. They now occur at about 22.5 and 24.1 mT, which is closer together than in Figure 2. Furthermore, the SW frequencies have shifted compared to those in Figure 3 when $R_{4}$ = 102 nm.

**Figure 5:**
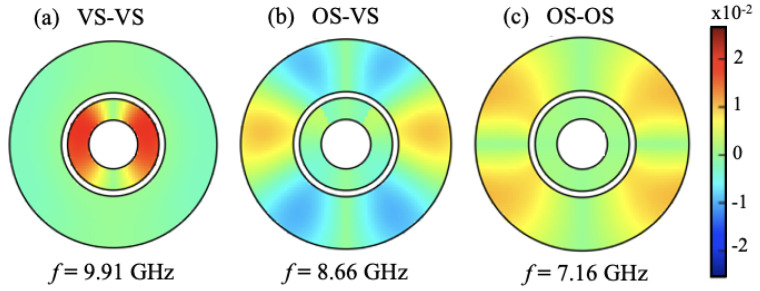
Color plots for the distribution of spin-wave amplitudes for nanorings of the same magnetic material (Py-Py) showing (**a**) VS-VS at $B_{0}$ = 0.01 T for mode 9, (**b**) OS-VS at $B_{0}$ = 0.015 T for mode 8, and (**c**) OS-OS at $B_{0}$ = 0.03 T for mode 6. The different colors provide information about the relative amplitude and phase.

In Figure 5, we show some examples for calculations of the spatial distribution of the SW mode amplitudes when the relative phase is included. Specifically, we display here the spatial (or areal) color plots for selected modes in each of the three stable configurations at different values of the applied magnetic field. The double ring structure is the same one as in Figure 3. As described in the theory section, the amplitudes at each spin site can be deduced from the eigenvectors of the dynamical matrix given in Equation (6). In the present application, it was found that the eigenvectors are real, but may be positive or negative (representing in-phase and $180^{\circ }$ out-of-phase oscillations, respectively). In the different cases shown here, it was seen that the amplitudes in the nanorings display both radial and circumferential variations.

### 3.2. Bicomponent Structures

**Figure 6:**
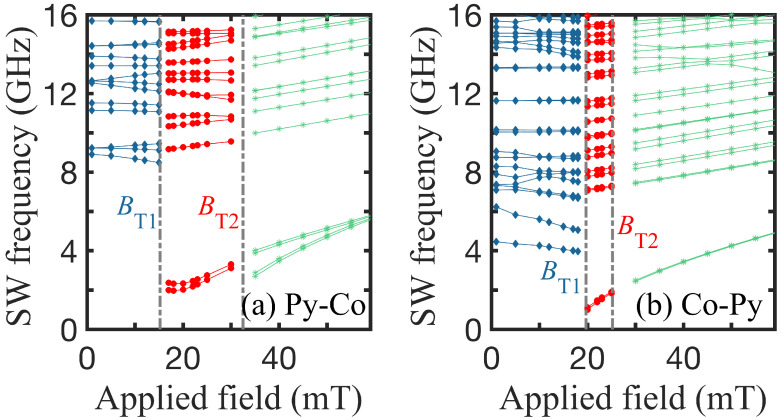
Lowest SW frequencies versus applied magnetic field showing the three stable regions for (**a**) Py-Co and (**b**) Co-Py nanorings. The radii in both cases are $R_{1}$ = 24 nm and $R_{2}$ = 48 nm, and $R_{3}$ = 54 nm and $R_{4}$ = 102 nm.

In this section, we present further results for concentric double nanorings in the case where the rings were composed of two different materials, chosen as Co and Py. The parameters for Py were as before while, for Co, the exchange stiffness was $D_{\mathrm{Co}}$ = 50.0 T nm^2^ , the saturation magnetization $M_{s}$ was such that $4\pi \mu_{0}M_{s}$ = 0.115 T, and the gyromagnetic ratio $g\mu_{B}$ = 31.0 GHz T^−1^, following [50]. Two distinct situations may arise, one in which ring A is made of Py and ring B made of Co (denoted as Py-Co), and the reverse situation in which ring A is made of Co and ring B made of Py (denoted as Co-Py). We may expect the SWs to display different characteristics in these two cases. In Figure 6, we compare the two situations of (a) Py-Co and (b) Co-Py, finding that the transition fields and SW frequencies are different. It is noticeable, in the Py-Co case, that there is a pronounced splitting between the two lowest branches in the high-field region, whereas this splitting effect is negligible in the Co-Py case. These differences are related to the fact that the exchange stiffness and saturation magnetization are significantly larger for Co. Thus, the outer part of the ring has a larger effect in the Py-Co case, and this is when the splitting is found to be more evident.

**Figure 7:**
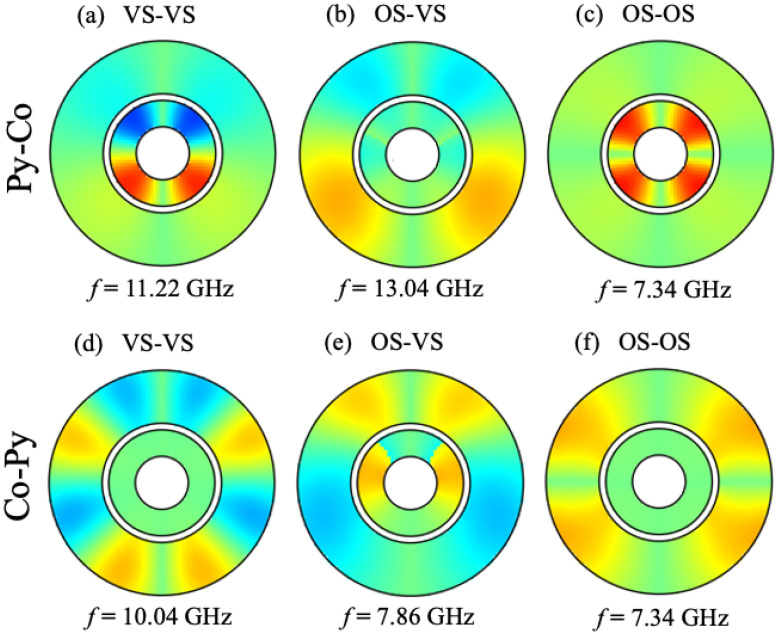
Color plots for the spatial distribution of SW amplitudes for nanorings composed of two different magnetic materials: Py-Co (top row) showing (**a**) VS-VS at $B_{0}$ = 0.01 T for mode 9, (**b**) OS-VS at $B_{0}$ = 0.015 T for mode 8, and (**c**) OS-OS at $B_{0}$ = 0.03 T for mode 6 and, for Co-Py (bottom row), (**d**) VS-VS at $B_{0}$ = 0.01 T for mode 8, (**e**) OS-VS at $B_{0}$ = 0.015 T for mode 6, and (**f**) OS-OS at $B_{0}$ = 0.03 T for mode 5. The colors provide information about the relative amplitude and phase (see the color bar given in Figure 5).

Next, in Figure 7, we show, as an example, some color plots for the spatial amplitude distribution with phase included for the SW modes calculated in both cases of the bicomponent nanorings (Py-Co and Co-Py). These respective distributions are seen to be distinctively different from one another, and, also, they differ in their stable configurations (VS-VS, OS-VS, and OS-OS), as shown in this example. By analogy with Figure 5, the radial and circumferential variations in amplitudes are seen to be prominent for both Py-Co and Co-Py nanorings.

**Figure 8:**
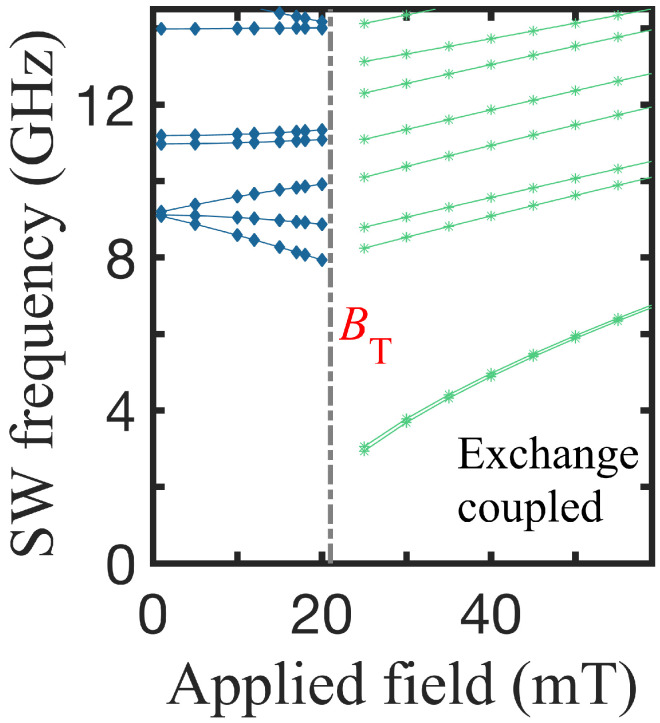
Lowest SW frequencies versus applied magnetic field for a Py-Co nanoring with no intermediate spacer and with exchange coupling across the interface (see the text). Under these conditions, there are only the VS-VS and the OS-OS regions. The radii are $R_{1}$ = 24 nm, $R_{2}$ = $R_{3}$ = 51 nm, and $R_{4}$ = 102 nm.

To date, we have studied situations in which the two nanorings are weakly coupled. By adjusting the spacer wall thickness, however, we may change the degree of inter-ring coupling. In Figure 8, the frequencies of the SW modes are shown as a function of the applied field for a Py-Co structure when there is no spacer (s= 0) between the two rings, i.e., there is a direct interface. For the exchange stiffness parameter across the interface between Py and Co, we assume an intermediate value given by the phenomenological expression $D_{I}={\left(D_{\mathrm{Py}}D_{\mathrm{Co}}\right)}^{1/2}$. Other choices could also have been made. In this situation of strong coupling between the component rings, we see that there is only a single transition occurring between the two stable VS-VS and OS-OS states (instead of a mixed state like VS-OS).

**Figure 9:**
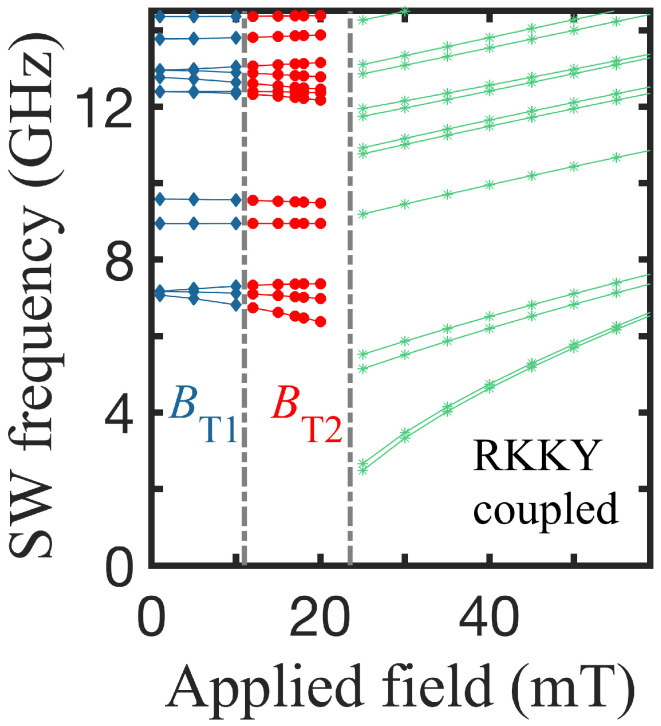
Lowest SW frequencies versus applied magnetic field showing the three stable regions for a Py-Co nanoring with an infinitesimal spacer width chosen to model a weak RKKY coupling (see the text). The radii are $R_{1}$ = 24 nm, $R_{2}$ ≈ $R_{3}$ = 51 nm, and $R_{4}$ = 102 nm.

Another case of interest occurs when the spacer radial width is small compared to our effective lattice parameter *a* (e.g., a Ru layer of order 1 nm or less), such that it nevertheless provides a Ruderman–Kittel–Kasuya–Yoshida (RKKY) coupling between the two rings (Py-Co). The behavior of the SW frequencies obtained in this case as a function of the applied field is shown in Figure 9. Since RKKY interactions are known to be relatively weak (and can either be positive or negative), we find that there may, again, be two transition fields and an intermediate OS-VS configuration state. Interestingly, we notice a relatively smooth behavior at $B_{T1}$ for the SW frequencies in this case. This contrasts, for example, with the behavior in Figure 6, for which there was no RKKY coupling. In this last example, we assumed for the RKKY coupling that $D_{\mathrm{RKKY}}/D_{\mathrm{Py}}=-0.005$.

## 4. Discussion

In summary, we have presented a theoretical analysis based on the microscopic Hamiltonian approach to study the static and dynamic magnetic properties of concentric double nanorings with a nonmagnetic spacer. Cases were considered where the rings were composed of the same or different magnetic materials. In the case of single-component nanorings, we studied the dependence of the mean spacer position on the two distinct transition fields and found that the first transition field decreased slightly with an increasing radial mean spacer position while the second transition field showed an increase. Areal color plots for the SW mode amplitudes displayed significant radial and circumferential differences between the inner and outer nanorings. In the bicomponent case, we observed that the two transition fields depended sensitively on whether the Py or Co was the inner ring. There was a consequent difference in behavior for the SW modes. We also demonstrated for a special case in which the spacer wall thickness was zero (s=0) that the strong interfacial exchange coupling resulted in there being only a single transition field. By contrast, however, the situation in which there was a very small spacer thickness *s* of a suitable material (e.g., Ru or Cr) giving a weak RKKY coupling, once again, led to there being two transition fields. Our results illustrate the rich variety of SW behaviors that are possible in these double nanoring structures; it would be interesting for them to be explored further experimentally using, e.g., micro-focused BLS. Although the best spatial resolution currently available with this technique is of the order of 200 nm, it is to be hoped that the ongoing improvements will continue. However, if some experimental results become available, there would be a justification to do more intensive computations for larger ring sizes (with, say, up to about $R_{4}$ = 500 nm for the outer radii). Another issue that would arise in calculations for larger ring sizes is that different possible magnetic states involving domain walls might arise (e.g., by analogy with [59,60]); these features would be interesting to explore in future work.

Some possible extensions of this work would be to study the SW modes in thicker samples of concentric double nanorings (e.g., where the overall thickness *t* becomes comparable with the radii of the nanorings) [61]. Alternatively, we could have vertically stacked concentric nanorings with similar or different feromagnetic materials for the two rings. In other examples, we could introduce the interfacial effects of Dzyaloshinskii–Moriya interactions [62,63,64,65,66] in these concentric rings. Extensions of our current work on concentric double nanorings to analyze concentric multiple (three or more) nanorings, as fabricated and characterized by Jain et al. [41], could be undertaken.

## Data Availability

All of the data present in this paper will be made available upon reasonable request. Please contact the corresponding author for further information.
